# Simultaneous profiling of seed-associated bacteria and fungi reveals antagonistic interactions between microorganisms within a shared epiphytic microbiome on *Triticum* and *Brassica* seeds

**DOI:** 10.1111/nph.12693

**Published:** 2014-01-21

**Authors:** Matthew G Links, Tigst Demeke, Tom Gräfenhan, Janet E Hill, Sean M Hemmingsen, Tim J Dumonceaux

**Affiliations:** 1 Agriculture and Agri-Food Canada Saskatoon Research CentreSaskatoon, SK, Canada; 2 Department of Veterinary Microbiology, University of SaskatchewanSaskatoon, SK, Canada; 3 Grain Research Laboratory, Canadian Grain CommissionWinnipeg, MB, Canada; 4 National Research Council CanadaSaskatoon, SK, Canada

**Keywords:** biocontrol, core microbiome, *cpn60* universal target, DNA barcode, epiphytes, seed microbiota

## Abstract

1. In order to address the hypothesis that seeds from ecologically and geographically diverse plants harbor characteristic epiphytic microbiota, we characterized the bacterial and fungal microbiota associated with *Triticum* and *Brassica* seed surfaces.
2. The total microbial complement was determined by amplification and sequencing of a fragment of chaperonin 60 (*cpn60*). Specific microorganisms were quantified by qPCR. Bacteria and fungi corresponding to operational taxonomic units (OTU) that were identified in the sequencing study were isolated and their interactions examined.
3. A total of 5477 OTU were observed from seed washes. Neither total epiphytic bacterial load nor community richness/evenness was significantly different between the seed types; 578 OTU were shared among all samples at a variety of abundances. Hierarchical clustering revealed that 203 were significantly different in abundance on *Triticum* seeds compared with *Brassica*. Microorganisms isolated from seeds showed 99–100% identity between the *cpn60* sequences of the isolates and the OTU sequences from this shared microbiome. Bacterial strains identified as *Pantoea agglomerans* had antagonistic properties toward one of the fungal isolates (*Alternaria* sp.), providing a possible explanation for their reciprocal abundances on both *Triticum* and *Brassica* seeds.
4. *cpn60* enabled the simultaneous profiling of bacterial and fungal microbiota and revealed a core seed-associated microbiota shared between diverse plant genera.

## Introduction

The seeds of crops such as wheat (*Triticum* spp.) and canola (*Brassica* spp.) are products of agricultural enterprise and the source of the next generation of plants. Thus healthy, high-quality seeds are critically important for the stability of the world’s food supply and the economic success of farmers. Crop seeds, like other parts of the plant, are colonized by an epiphytic microbiota consisting of synergistic, commensal and potentially pathogenic microbes that play a crucial role in health and susceptibility to disease (Hashidoko, 2005; Critzer & Doyle, 2010). Because the plant-associated microbiota clearly plays a role in plant fitness (Hallmann *et al*., 1997), different crops might be expected to harbor distinct microbiota on their seed surfaces and the constituents of these microbial communities are likely to have functional relevance during plant growth, development and seed storage. For example, specific microorganisms such as *Penicillium verrucosum* and *Alternaria alternata* in stored crop seeds can cause spoilage, decreasing crop value, or produce mycotoxins that have a direct effect on human health (Magan & Aldred, 2007; Duarte *et al*., 2010; Magan *et al*., 2010). Conversely, commonly utilized crop rotations, such as canola-wheat, are known to have positive benefits for yields and for pathogen control (Zegada-Lizarazu & Monti, 2011; Bushong *et al*., 2012; Harker *et al*., 2012). Microorganisms that associate with each crop may influence the growth and development of the subsequent crop in the rotation. The potential impact of crop-based microbial communities on yields and on pest control demands that a comprehensive knowledge of microbiota associated with seed surfaces be elucidated.

Microorganisms – both bacteria and fungi – that associate with plants can have beneficial, neutral or harmful effects on the host. Well-known examples of beneficial plant–microbe interactions include symbioses such as nitrogen fixation by root-associated Rhizobiales (Hayat *et al*., 2010), as well as antagonism toward potential pathogens by plant-associated microorganisms (Braun-Kiewnick *et al*., 2000). It is becoming increasingly appreciated that microbes are intimately associated with plants and can influence host growth and metabolic processes. For example, various aseptically cultured *Atriplex* spp. were shown to have a variety of root- and leaf-associated microbes, including both bacteria and fungi, that probably originated as seed endophytes (Lucero *et al*., 2011). Furthermore, the epiphytic phyllosphere microbial community on *Lactuca sativa* consists of a core microbiota consisting of *Pseudomonas*, *Bacillus*, *Massilia*, *Arthrobacter* and *Pantoea* spp., and the abundance of genera such as *Pantoea* correlates negatively to pathogen abundance, suggesting possible antagonistic interactions between foliar microbiota and potential pathogens (Rastogi *et al*., 2012). An understanding of the composition and dynamics of the plant-associated microbiota, which is facilitated by modern sequencing technologies, is expected to lead to agronomic benefits including enhanced plant growth and the identification of microorganisms that inhibit the growth of potential pathogens (Tikhonovich & Provorov, 2011).

Seed-borne endophytes have been shown to be an important source of bacteria within other tissues (Hallmann *et al*., 1997; Lucero *et al*., 2011). The identification of a set of endophytic microbes among *Zea* spp. that are conserved across evolutionary and ecological boundaries (Johnston-Monje & Raizada, 2011) suggests that plants select microbes from the environment with beneficial properties to the host plant. By contrast, seed-associated epiphytes, microorganisms associated with the seed surface, have been relatively uncharacterized. The organisms that naturally associate with seed surfaces may be expected, like seed-associated endophytes and foliar epiphytes, to have been selected from the environment by the host for mutualistic interactions such as the inhibition of pathogen growth. If that is true, then different plant hosts might harbor a characteristic set of microbes on their seed surfaces and the microbial profile obtained from the seeds of a particular plant may be exploited as a diagnostic tool to determine the source and quality of seed stocks. We tested the hypothesis that the seed-associated epiphytic microbiota of two taxonomically diverse crop plants, *Triticum* spp. (wheat) and *Brassica* spp. (canola/mustard) are distinguishable. Furthermore, by comparing the assembled OTU sequences with those from bacteria and fungi isolated from these samples we demonstrated that OTU could be assembled *de novo* with 100% identity to microbes in complex samples. Finally, we examined the interactions of microorganisms that were originally identified based on microbial profiling and identified strains of *Pantoea agglomerans*, a numerically dominant seed epiphyte, that can inhibit the growth of potentially pathogenic and seed spoilage-associated microorganisms.

## Materials and Methods

### Seed sources

Crop seeds of diverse geographic origins within Canada were chosen for analysis. Seeds of *Brassica* (*B. juncea* L. Czern., *B. rapa* L., *B. napus* L.) and *Triticum* (*T. aestivum* L., *T. turgidum* L. subsp. *durum* (Desf.) Husn.) were used for the study. In total, five replicates of seed-surface microbiota were analyzed for *Brassica* and six replicates for *Triticum* were sequenced (Table1). The seeds were harvested in the summer of 2008 and the experiment was carried out in 2009. The seeds were stored separately in plastic bags at room temperature for *c*. 12 months before DNA extraction.

**Table 1:** Description of samples

Sample name	Sample source	Sample description^1^	Geographic origin^2^
Wheat-1	*T. turgidum* L. subsp. *durum* (Desf.) Husn.	CWAD, grade 2	Western Canada
Wheat-2	*T. turgidum* L. subsp. *durum* (Desf.) Husn.	CWAD, grade 3	Western Canada
Wheat-3	*Triticum aestivum* L.	CESRW, grade 2	Eastern Canada
Wheat-4	*Triticum aestivum* L.	CWRS, grade 1	Western Canada
Wheat-5	*Triticum aestivum* L.	CWRS, grade 2	Western Canada
Wheat-6	*Triticum aestivum* L.	CWRS, grade 3	Western Canada
Brassica-1	*Brassica juncea* (L.) Czern.	Brown mustard, grade 1	Western Canada
Brassica-2	*Brassica napus* L.	Canola B	Western Canada
Brassica-3	*Brassica napus* L.	Canola A	Western Canada
Brassica-4	*Brassica juncea* (L.) Czern.	Oriental mustard, grade 1	Western Canada
Brassica-5	*Brassica rapa* L.	Brown mustard	Western Canada

CWRS, Canada Western Red Spring wheat; CESRW, Canada Eastern Soft Red Winter wheat; CWAD, Canada Western Amber Durum wheat.

Seeds were sourced from different geographic locations in Eastern Canada (Ontario or Quebec) or Western Canada (Manitoba, Saskatchewan, Alberta, or British Columbia).

### DNA extraction from seed-associated epiphytic microbiota

A 10 g sample of each seed lot was soaked in a solution of 45 ml buffered peptone water (10 g peptone, 5 g NaCl, 3.5 g Na_2_HPO_4_, 1.5 g KH_2_PO_4_ l^−1^; Kim *et al*., 2006) containing 0.05% Triton X-100 (Sigma) in a 250 ml Erlenmeyer flask at room temperature with shaking (150 rpm) for 1 h. The liquid fractions were centrifuged at 4000 ***g*** for 15 min and the supernatant discarded. Pellets were resuspended in 200 μl of TE buffer and subjected to DNA extraction using the previously described bead-beating protocol (Hill *et al*., 2005). DNA was quantified using a Quant-IT DNA quantification kit and Qubit fluorometer (Invitrogen).

### Quantification of bacterial *16S* rRNA-encoding genes

Due to higher sequence conservation of the *16S* rRNA primer landing sites, we determined the total bacterial load in each seed lot by quantitative PCR of bacterial *16S* rRNA-encoding genes using the universal primers SRV3-1 and SRV3-2 (Lee *et al*., 1996). Reactions were prepared using SsoFast EvaGreen supermix (Bio-Rad) with 400 nM of each primer in a final volume of 20 μl. Amplification conditions were: 95°C for 3 min (1 ×); followed by 30 cycles of 95°C for 15 s, 62°C for 15 s, and 72°C for 15 s. Data collection was set at the extension step. Results were expressed as *16S* rRNA gene copies g^−1^ seeds by considering the weight of seeds used for extraction and the template volume used for qPCR.

### *cpn60* universal target (UT) amplicon generation and sequencing

Amplicons were generated from each sample using multiplexing ID (MID)-adapted universal primers as described previously (Schellenberg *et al*., 2009, 2011). Purified, concentrated amplicon from all seed samples was pooled on an equimolar basis before emPCR adaptor ligation and pyrosequencing using Titanium chemistry (Roche/454).

### Assembly of operational taxonomic units

The de-multiplexing of pyrosequencing data was done as described previously (Chaban *et al*., 2012). mPUMA (Links *et al*., 2013) was used with default parameters to process de-multiplexed data using *de novo* assembly (via gsAssembler) for OTU formation and mapping of reads via bowtie2 (Langmead & Salzberg, 2012) for determination of OTU abundance.

### α-diversity measures

In order to avoid biases introduced by unequal sampling effort (Gihring *et al*., 2012), OTU abundance data for each sample was sub-sampled at random to the size of the smallest library (3606 reads). Calculation of community parameters including Chao1 richness, Simpson’s index D, the Shannon–Weiner index (H′) and Good’s coverage estimator was performed using mothur (Schloss *et al*., 2009).

### Analysis of OTU abundance across crops

**Figure 1:**
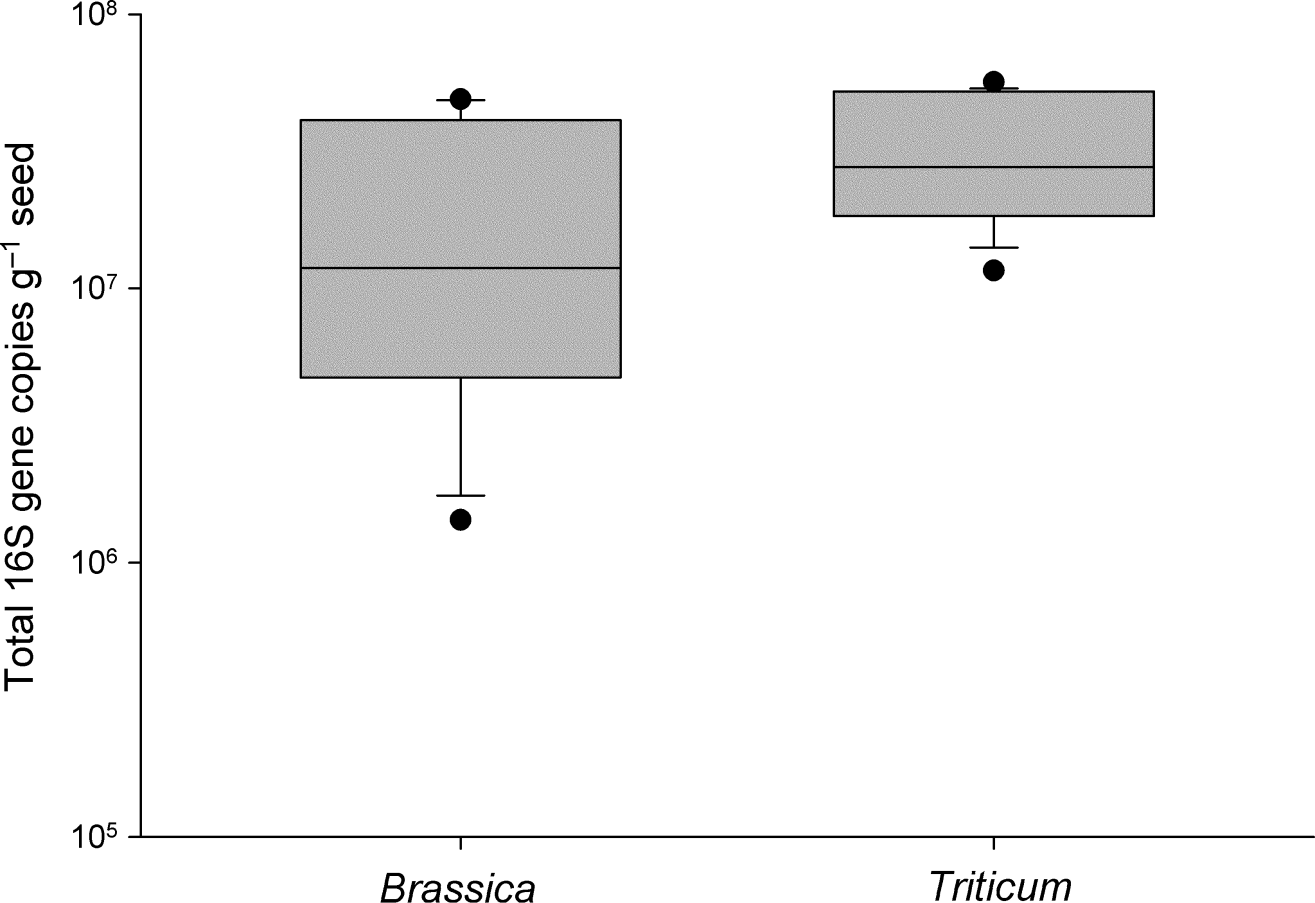
Total bacterial *16S* rRNA gene copies g^−1^ seed as measured by quantitative PCR for *Brassica* and *Triticum* seed washes. The lower and upper edges of each box correspond to the 25th and 75th percentiles, while the whiskers correspond to the 10th and 90th percentiles. Dots indicate outliers. The median value is indicated by a horizontal line.

Before analysis in R (http://www.r-project.org) the OTU abundances were scaled to a total library size of 10^7^ to approximate the community size as measured by *16S* rRNA copies g^−1^ for these samples (Fig.1). Clustering and statistical tests based on OTU abundance were performed in R (v2.15.1) on a Linux server (CentOS 5.8). Hierarchical clustering was performed using an average linkage method based on the Euclidean distance of both OTU and samples. OTU with significantly differential abundances were identified using an unpaired Mann–Whitney test followed by a Benjamini–Hochberg correction for multiple hypothesis testing at an alpha = 5% level of significance.

### Quantitative PCR targeting specific microbes

Primers designed to target specific OTU were designed using sigoligo (Zahariev *et al*., 2009), Beacon Designer 7 (Premier Biosoft, Palo Alto, CA, USA), and primer3 (Rozen & Skaletsky, 2000). Primers targeting bacterial OTU00845 were 5′-CGG TAT TGA CCA GGC TGT TAT C-3′ and 5′-AGT TCA ATC GCA CCG GTT T-3′ (271 bp product). Amplification conditions used were 95°C for 3 min followed by 40 cycles of 95°C for 15 s, 60°C for 15 s, and 72°C for 30 s. Primers targeting fungal OTU03024 were 5′-GCT TGA GGT TAC CGA AGG-3′ and 5′-GGA GAG GAG GAT CAG AGG-3′ (112 bp product). Amplification conditions were 95°C, 3 min followed by 40 cycles of 95°C, 15 s, 63°C, 15 s, 72°C, 30 s. For both assays, data collection was at the extension step (72°C). Quantitative PCR with SsoFast Eva Green Supermix (Bio-Rad) and primer concentrations of 400 nM each was used to determine the apparent genome number of each organism in each seed extract as described (Dumonceaux *et al*., 2006).

### Isolation and identification of microbes

In order to isolate fungi from *Brassica* or *Triticum* seeds, a 4 g sample of seeds was incubated in 50 ml of Taylor minimal medium (Taylor, 1993) and on malt extract broth (Difco, Houston, TX, USA), each containing antibiotics: tetracycline (100 μg ml^−1^); streptomycin (100 μg ml^−1^); and penicillin (1000 units ml^−1^). Seed samples were incubated with shaking (150 rpm) at room temperature (20–23°C) for 4 d, then 100 μl of serial dilutions of the broth were plated on Taylor minimal medium and on malt extract agar plates with antibiotics until colonies appeared. Some samples showed outgrowth in broth culture of large mycelial agglomerates; these were blended in a sterile Eberbach blender cup for 10 s before dilution and plating. A similar strategy was used to isolate bacteria from *Triticum* seeds, except that 50 ml of antibiotic-free trypticase soy broth (Difco) was used as a culture medium and the cultures were incubated overnight at room temperature before dilution and plating on trypticase soy agar plates. DNA was extracted from each fungal strain using a miniprep method (Wendland *et al*., 1996) and from each bacterial strain using a Wizard genomic DNA extraction kit (Promega). The *cpn60* UT sequences of bacterial isolates were determined by direct sequencing of amplicons using M13-adapted universal primers H729/H730 as described previously (Goh *et al*., 2000). Sequences of the nuclear ribosomal internal transcribed spacer (ITS) were determined for each fungal isolate using PCR primers and amplification conditions as described (Schoch *et al*., 2012).

### Phylogenetic analysis

Full-length assembled OTU sequences were aligned with the *cpn60* sequences determined from the isolates as described above and with selected reference strains from cpnDB (http://www.cpndb.ca) using ClustalW (Thompson *et al*., 1994). Phylogenetic trees were constructed using the neighbor-joining method (Saitou & Nei, 1987) with bootstrapping of 500 replicates. Distances were calculated using the maximum composite likelihood method. Alignments were performed and trees were calculated using MEGA v5.05 (Tamura *et al*., 2007).

### Biological interaction assays

*Triticum* seeds (Canada Western Red Spring wheat, grade 3) were sterilized by submerging 30 g of seeds within a nylon bag in 250 ml of 95% ethanol for 20 s, followed by 250 ml of 20% commercial bleach for 15 min with shaking. Seeds were then washed in 7 × 250 ml of sterile water (3 min for the first three washes and 10 min for the final four washes). Sterilized seeds were dried overnight in a sterile Petri dish. Seeds were re-colonized with the desired strains by diluting overnight cultures of each strain 1 : 100 in 5 ml of sterile peptone water, then adding *c*. 50 seeds to each dilution. This inoculum corresponded to *c*. 1.6 × 10^7^ CFU g^−1^ seeds. Control seeds were added to sterile peptone water without bacterial culture. The seeds were incubated at room temperature for 15 min with gentle agitation, and then placed in the center of plates containing Czapek-Dox agar medium (containing 30 g sucrose, 2 g sodium nitrate, 1 g dipotassium phosphate, 0.5 g each of MgSO_4_ and KCl, and 0.01 g of FeSO_4_ l^−1^). A 5-mm punchout from the edge of a colony of *Leptosphaeria maculans* strain WA51 (Yu *et al*., 2005) or of fungal isolate 15 was placed within 3 cm of the seeds and the plates were incubated at 25°C for 1 wk. Inhibition of fungal growth was scored using previously described methods (Chakraborty *et al*., 1994).

## Results

### Total *16S* rRNA-encoding gene counts

The total *16S* rRNA gene copy number associated with each *Triticum* seed type varied over a range of *c*. 4-fold, with Wheat-4 (CWRS grade 1) being the lowest and Wheat-2 (CWAD grade 3) the highest (Fig.1). The range was somewhat wider (*c*. 9-fold) within the *Brassica* seeds, with Brassica-5 (*B. rapa*) being the lowest and Brassica-4 (oriental mustard) the highest. Although the *Triticum* samples tended to have higher *16S* rRNA gene counts than the *Brassica* samples, no statistically significant differences were detected at a significance level of 0.01 (*P* = 0.018, Mann–Whitney rank sum test).

### Pyrosequencing of *cpn60* UT amplicons

A total of 408 658 reads was generated from the 11 amplicon libraries. The median library size was 34 594 with a range of 3606 reads (*B. rapa*) to 96 834 reads (CWRS grade 3). These reads were assembled into 5477 distinct OTU. Rarefaction plots as well as Family-level taxonomic profiles of the *Brassica* and *Triticum* samples are provided in Supporting Information Notes S1 and Fig. S1.

### Microbial community diversity

Community richness (Chao1, expressed as the projected total number of OTU in each sample), evenness (Simpson’s index, D) and the Shannon index H′ (Hill *et al*., 2003) was calculated for each sample. No correlation was observed between community richness or evenness, and total bacterial *16S* rRNA gene copy numbers (Spearman rank correlation; not shown). Comparing the microbial communities associated with *Triticum* (*n* = 6) and *Brassica* (*n* = 5) revealed no significant differences in the diversity parameters by Mann–Whitney test and one-way ANOVA (Fig. S2).

### The shared epiphytic microbiota of *Triticum* and *Brassica* seeds

Microbial profiles determined for *Triticum* and *Brassica* samples were compared, resulting in the identification of a core microbiome for each host plant genus. All *Triticum* (*n* = 6) samples had 262 OTU in common while all *Brassica* (*n* = 5) samples had 215 OTU in common. In order to identify the microbiota shared between seeds of *Brassica* and *Triticum* we established a sample prevalence of at least 7/11 as a lower limit for an OTU to be considered shared. This would ensure that any OTU identified as shared was observed in at least one sample of each host genus. There were 578 OTU identified with a sample prevalence of 7/11 or higher. Additionally we determined whether there were any OTU found in all samples. Across host plant species 64 OTU were detected in every sample. We examined the effect of sample size (per host plant genus) on the number of OTU identified as shared. The number of shared OTU was calculated for each combination of *Brassica* and *Triticum* samples (from 1 to 5 samples for each host plant genus). The number of shared OTU diminished as sample size increased in a nonlinear fashion, suggesting an asymptote around 60 OTU (Fig. S3). These results are consistent with the identification of a shared microbiome at the sample size used in this study and suggest that larger sample numbers would not substantially decrease the size of the shared microbiome.

Sequences for these 64 shared OTU were similar but not identical (88–99% identity) to records from cpnDB that included *Pantoea agglomerans*/*Erwinia herbicola* (99%), *Massilia timonae* (93%), *Pantoea stewartii* (93%), *Porphyrobacter sanguineus* (88%), *Pseudomonas fluorescens* (97%), *Pseudomonas syringae* (95%), *Pyrenophora tritici-repentis* (93%), *Sphingobium japonicum* (90%), *Sphingomonas wittichii* (90%)*, Telluria mixta* (93%), *Xanthomonas axonopodis* (94%), *Xanthomonas fuscans* (95%) and some novel sequences.

### Differential OTU abundances

**Figure 2:**
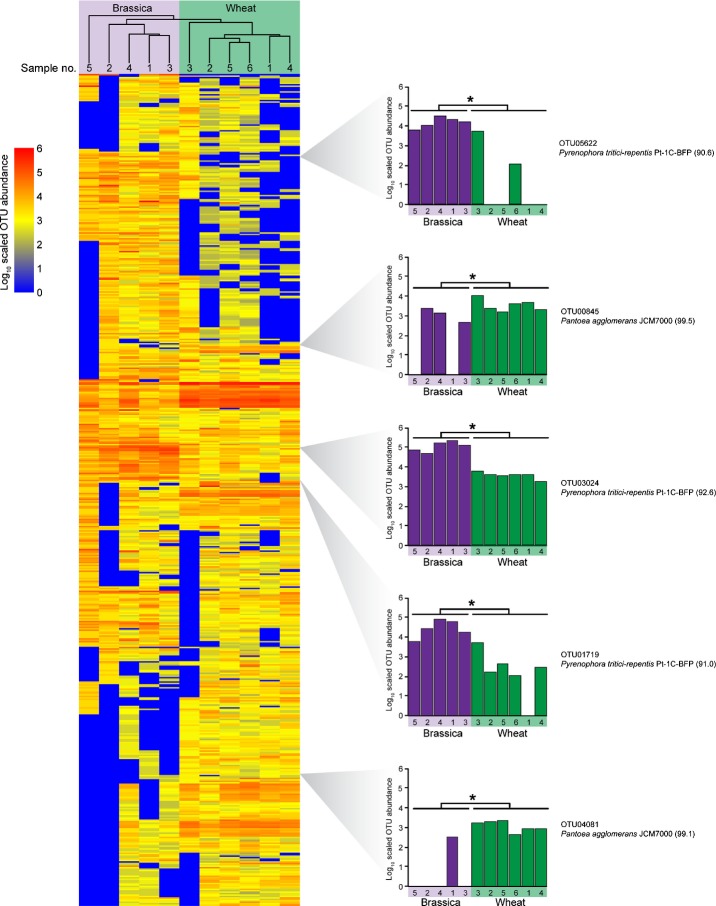
Hierarchical clustering of samples and operational taxonomic units (OTU) from crop seeds. These 578 OTU were found in at least 7/11 samples from the two seed types. Libraries are represented by columns while OTU are represented by rows. The abundances of all OTU in each column (sample) sum to a total scaled library size of 10^7^ and are presented as a heat map (blue, less abundant to red, more abundant). Specific OTU corresponding to cultured isolates are identified along with their corresponding read abundances in each library and cpnDB (http://www.cpndb.ca) nearest neighbor (with percentage identities indicated in parentheses). Asterisks indicate statistically significantly different abundances measured by sequencing read counts (unpaired Mann–Whitney test with Benjamini–Hochberg correction for multiple hypothesis testing at α = 5% level of significance).

Hierarchical clustering of the microbial profiles showed that the *Triticum-* and *Brassica*-derived samples could be separated on the basis of the 578 shared OTU (Fig.2). A Mann–Whitney test identified 203 of these OTU that were significantly differentially abundant between *Triticum* and *Brassica,* including all 64 OTU with a sample prevalence of 11/11 (Table S1).

Approximately 40% of the significantly differentially abundant OTU (79/203, including OTU00845) were closely related (96–99% nucleotide identity) to *P. agglomerans*. *Triticum* seeds were found to have significantly more sequences from these *Pantoea*-like OTU than *Brassica* seeds (Table S1). Fungal OTU were also identified as significantly different in abundance between *Brassica* and *Triticum* samples with 12 OTU (including OTU03024) more abundant on *Brassica* seeds as compared with the *Triticum* seeds. While the cpnDB nearest neighbor for these OTU was *Pyrenophora tritici-repentis* (*c*. 94% identity), these fungal OTU were more similar (up to 99% identity) to a truncated *cpn60* UT sequence from *Alternaria alternata* (GenBank GQ871196).

**Figure 3:**
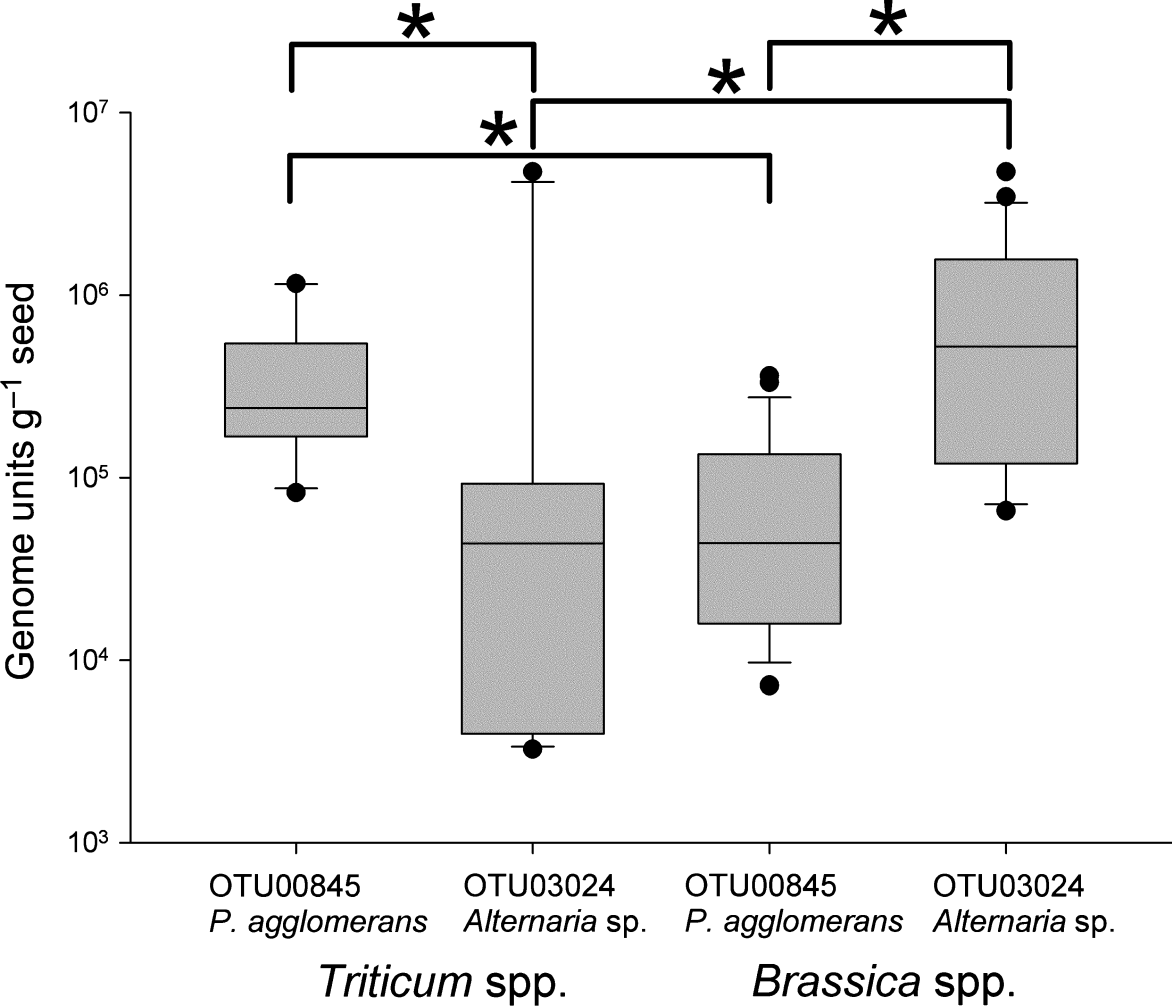
Quantification by qPCR of OTU00845 (*Pantoea agglomerans*) and OTU03024 (*Alternaria* sp.) on seeds of *Triticum* (*n* = 12) and *Brassica* (*n* = 24). qPCR results are from at least two biological replicates (DNA extractions) and two technical replicates per sample. The lower and upper edges of each box correspond to the 25th and 75th percentiles, while the whiskers correspond to the 10th and 90th percentiles. Dots indicate outliers. The median value is indicated by a horizontal line. Significant differences in the median values measured by the Mann–Whitney rank-sum test (*P* < 0.01) are indicated (*).

Quantitative PCR assays targeting OTU00845 and OTU03024 validated the sequence read abundance patterns seen in the microbial profiles of *Triticum* and *Brassica* (Fig.3). Consistent with the sequencing read counts, the *P. agglomerans* OTU were significantly more abundant on *Triticum* seeds than *Brassica,* while the *Alternaria*-like OTU03024 exhibited an inverse pattern, being more abundant on *Brassica* seeds (Fig.3).

### Isolation of bacteria and fungi from *Triticum* and *Brassica* seeds

**Figure 4:**
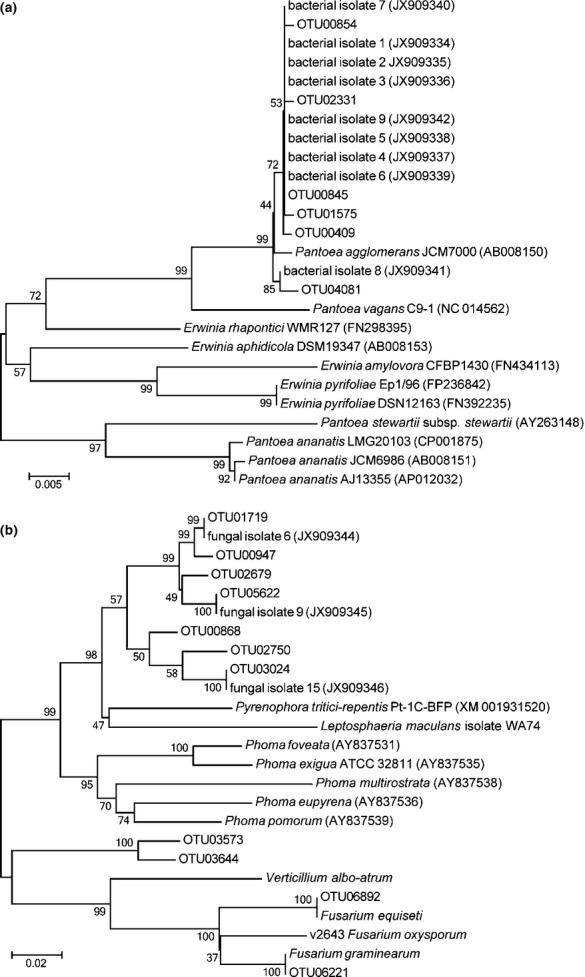
Phylogenetic analysis of the *cpn60* UT sequences of selected operational taxonomic units (OTU) assembled from pyrosequencing data along with reference strains from cpnDB and isolates from *Triticum* and *Brassica* seeds. In both (a) and (b), the robustness of each node is indicated by the percentage of 500 trees in which the associated taxa cluster together and is presented next to the branches (Tamura *et al*., 2004). The scale bar represents units of base substitutions per site. Sequences corresponding to the *cpn60* UT of reference strains were retrieved from cpnDB with the nucleotide accession number (http://ncbi.nlm.nih.gov) for each strain indicated in parentheses. (a) *Pantoea agglomerans*-related OTU, reference strains, and isolate sequences. (b) Fungal isolates and OTU along with reference strain sequences from cpnDB.

In order to assess potential interactions between members of the shared microbiota, we undertook efforts to culture bacteria corresponding to *Pantoea-*like OTU00845 and fungi corresponding to *Alternaria*-like OTU03024. These specific OTU were targeted due to their reciprocal patterns of abundance in both the microbial profiling and qPCR results (i.e. on seeds where *Pantoea*-like OTU were abundant, *Alternaria*-like OTU were lower and vice versa; Fig.3 and Table S1). Multiple bacterial colony morphologies were observed when *Triticum* seeds were incubated in trypticase soy broth. Nine yellow colonies were picked from these plates, and these yielded a band with the *P. agglomerans cpn60* UT-specific primer set and were sub-cultured to purity. Microscopic analysis revealed that the organisms were Gram-negative rods and they formed yellow colonies on the trypticase soy agar plates, consistent with previous reports for *Pantoea agglomerans* (Lee & Liu, 1991). Determination of the *cpn60* UT sequences for all nine isolates revealed > 99% sequence identity with *P. agglomerans* JCM7000 and that eight of these were 100% identical with each other, and OTU00845. The isolate sequences clustered together with the OTU sequences and all were distinct from the *P. agglomerans* reference strain as well as from other *Erwinia* and *Pantoea* species (Fig.4a).

Fungi were also isolated from *Brassica* and *Triticum* seeds, with a wide range of colony morphologies observed, including yeasts, molds and filamentous phenotypes. Fungal isolates 6, 9 and 15 had *Alternaria*-like colony morphology and their *cpn60* UT sequences were identical to OTU01719, OTU05622 and OTU03024, respectively (Fig.4b). The *cpn60* UT sequence of a fourth isolate with similar morphology (fungal isolate 5) was 1 bp different from nonsignificant OTU02724 in a short homopolymer (not shown). The *cpn60* UT sequences of fungal isolates 5, 6 and 9 (and of OTU 02724, 05622 and 01719) shared 96–99% identity over 483 bp with a truncated *cpn60* UT sequence from *Alternaria alternata* (GenBank: GQ871196). The *cpn60* UT sequence of fungal isolate 15 was distinct from the other isolates (92% identity) and identical to OTU03024. The nuclear ribosomal internal transcribed spacer (ITS) region is a recently proposed DNA barcode marker for Fungi (Schoch *et al*., 2012), and we examined the ITS sequences of the isolates to place them in the context of known fungal strains. This analysis suggested that isolates 5, 6 and 9 were most closely related to *Alternaria alternata* while isolate 15 clustered with *Alternaria infectoria* and *Alternaria triticina* (Fig. S4). These observations were consistent with the morphological features of the fungal conidia, which were also typical of *Alternaria* spp. (data not shown).

### Interactions between bacterial and fungal isolates

**Figure 5:**
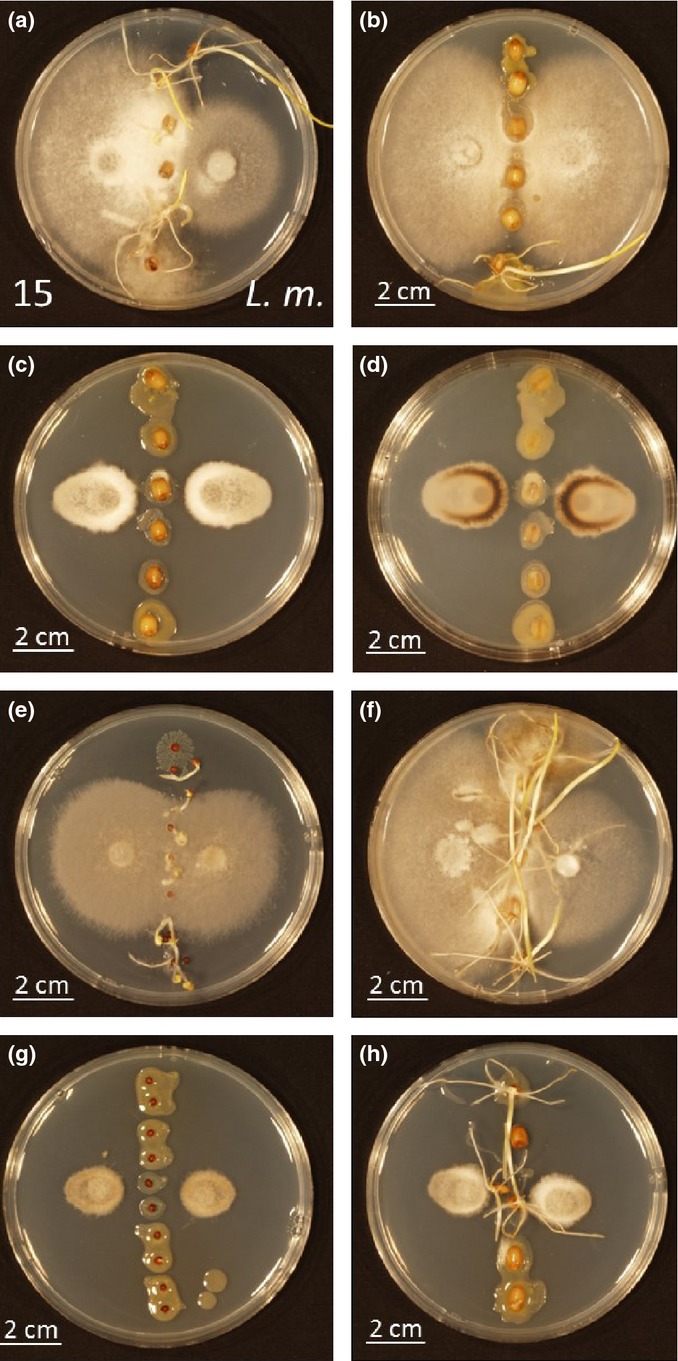
Interactions between selected bacterial isolates and fungal isolate 15. In some instances (a, b, e, f, h), the seeds have begun to germinate, producing shoots on the plates. (a) Sterilized wheat seeds not re-colonized with bacteria. Fungal isolate 15 is inoculated on the left while *Leptosphaeria maculans* (*L.m*.) is on the right. In all of the remaining panels, fungal isolate 15 is inoculated on the left and right sides of the seeds. (b) Sterilized wheat seeds colonized with bacterial isolate 1 (homogenous). (c) Same as (b) but with bacterial isolate 4 (aversion) – top view. (d) Same as (c) – bottom view. (e) Nonsterilized canola seeds. (f) Nonsterilized wheat seeds. (g) Nonsterilized canola seeds colonized with bacterial isolate 4. (h) Nonsterilized wheat seeds colonized with bacterial isolate 4.

The nine *P. agglomerans* isolates from *Triticum* seeds showed a spectrum of growth suppression against fungal isolate 15 (*Alternaria* sp.; identical to OTU03024) (Fig.5) as well as the canola blackleg pathogen *Leptosphaeria maculans* (Fig. S5). *Pantoea agglomerans* isolate 4 (identical to OTU00845) showed the strongest inhibition while other strains (isolates 3, 6 and 8) as well as unsterilized and sterilized seeds showed no inhibition on both wheat and canola (Table2; Figs5, S5). Some of the strains resulted in growth cessation of *L. maculans* at the point of contact, but fungal growth continued away from the bacterial colony (Table2; Fig. S5). In general the inhibition of *L. maculans* growth was stronger than of fungal isolate 15 (*Alternaria* sp.) by several of the isolates, but isolate 4 (identical to OTU00845) was quite effective against both fungi (Table2; Figs5, S5). Fungal isolate 15 produced a dark pigment upon interaction with bacterial isolate 4 and limited growth continued only in the direction opposite the bacterial challenge (Fig.5). Colonization of wheat and canola seeds with bacterial isolate 4 protected both seed types from overgrowth of both the *Alternaria*-like strain (Fig.5) and *L. maculans* (Fig. S5) in these assays.

**Table 2:** Interactions between bacterial isolates and *Leptosphaeria maculans* or fungal isolate 15

Bacterial isolate	Colony diameter increase per day, (mm)[Table-fn tf2-1]		Interaction[Table-fn tf2-2]	
	*L. maculans*	Fungal isolate 15	*L. maculans*	Fungal isolate 15
Isolate 1	4.87 ± 0.47	6.84 ± 1.85	Growth cessation	Homogenous
Isolate 2	4.31 ± 0.12	6.63 ± 1.78	Growth cessation	Homogenous
Isolate 3	3.83 ± 0.35	6.65 ± 2.06	Homogenous	Homogenous
Isolate 4	0.24 ± 0.38	1.20 ± 0.36	Aversion	Aversion
Isolate 5	4.69 ± 0.79	6.74 ± 1.88	Growth cessation	Homogenous
Isolate 6	4.64 ± 0.43	6.63 ± 2.18	Homogenous	Homogenous
Isolate 7	4.76 ± 0.70	5.63 ± 1.21	Growth cessation	Homogenous
Isolate 8	4.58 ± 0.50	7.28 ± 1.92	Homogenous	Homogenous
Isolate 9	4.32 ± 0.49	6.84 ± 1.41	Growth cessation	Homogenous
Sterile seed	4.90 ± 0.87	6.81 ± 1.95	Homogenous	Homogenous
Nonsterile seed	5.08 ± 0.48	7.42 ± 2.13	Homogenous	Homogenous

Colony diameter measurements are the mean of 4 or 6 measurements ± SD.

Interactions were scored according to Chakraborty *et al*. (1994).

## Discussion

The microbial complement that is naturally associated with multicellular organisms plays an important role in host health and disease. In plants, the importance of the epiphytic microbiota associated with different plant surfaces is beginning to be appreciated (Rastogi *et al*., 2012), but few studies have examined the composition of the seed-associated epiphytic microbiome. A comprehensive analysis of the microbial community that colonizes seeds requires a method that can detect and differentiate a wide variety of microorganisms in a single sample, which can be accomplished by taxonomic profiling using molecular markers. Taxonomic profiling typically exploits the *16S* rRNA-encoding gene, which is a very useful phylogenetic marker due to its universality and ability to discriminate closely related organisms (Schloss *et al*., 2011). Indeed, the depth and breadth of the database associated with *16S* rRNA sequences (RDP), as well as the sophisticated tools that have been developed for characterizing microbial communities profiled with this taxonomic marker (Schloss & Handelsman, 2008), have led to its widespread use for gene-centered metagenomic studies of bacterial communities. Despite its advantages, known limitations such as its variable copy number, comparatively low sequence divergence among closely related taxa, and lack of consistency in the domains examined across different studies can limit the utility of the information provided by *16S* rRNA sequence analysis. An alternative molecular taxonomic marker, the *cpn60* UT, possesses all of the necessary features of a marker for phylogenetic analysis of microbial communities. This protein-encoding gene is present in nearly all organisms including prokaryotes (with the exception of certain Mollicutes) and eukaryotes where it functions as a molecular chaperone assisting in the formation and maintenance of protein structures (Hemmingsen *et al*., 1988). Moreover, the sequence of the *cpn60* UT is accessible with a set of PCR primers (Hill *et al*., 2006) that can simultaneously amplify the region from both bacteria and fungi; in this sense *cpn60* offers a fundamental advantage over the *16S* rRNA-encoding gene. Furthermore, *cpn60* UT sequence identities are robust predictors of genome-scale sequence identities (Verbeke *et al*., 2011), while *16S* rRNA sequences are poor predictors (Zeigler, 2003). Many studies have demonstrated the ability of *cpn60* UT sequences to differentiate closely related organisms (Goh *et al*., 2000; Vermette *et al*., 2009), and *cpn*60 has been shown to be a preferred DNA barcode for bacteria (Links *et al*., 2012). The uniform length of the *cpn60* UT (nearly always ± 1 codon of 555 bp; Hill *et al*., 2004) makes sequence alignments trivial using classical methods. Known disadvantages of using the *cpn60* UT include the primer degeneracy and the necessity to amplify targets with a cocktail of primers at a range of annealing temperatures to profile all community members (Hill *et al*., 2006). Furthermore, the RDP has an enormous representation of divergent taxa from a wide array of environments compared with the analogous chaperonin database, cpnDB. Nevertheless, the *cpn60* UT offers a viable complementary tool to *16S* rRNA-encoding sequences for bacterial community profiling with specific advantages and disadvantages that can be exploited for various experiments.

In this work, we used the *cpn60* UT to describe similarities and differences in the microbiota associated with *Brassica* and *Triticum* seed surfaces and to determine whether *de novo* assembly of OTU (Links *et al*., 2013) provides accurate molecular barcodes for these microbial communities. In 13 separate cases, including both bacteria and fungi, the assembled OTU sequences were identical (*n* = 11) or nearly identical (99%; *n* = 2) to those obtained from isolates. This demonstrates that *de novo* assembly of OTU yields biologically relevant sequence barcodes that can be used for specific molecular diagnostic assays to detect and quantify microorganisms using established techniques (Dumonceaux *et al*., 2006, 2009). This is a particularly significant advantage in cases where an OTU sequence is assembled with little similarity to available reference sequences.

Examination of the microbial communities associated with seeds of these diverse plant species revealed a total epiphytic microbial load of *c*. 10^6^–10^8^ bacterial genomes g^−1^ seeds. Because the analysis of endophytic communities requires harsh treatment such as trituration or sonication of the plant material (Hallmann *et al*., 1997; Lundberg *et al*., 2012), the microbial communities we analyzed by gently washing the seeds likely represents predominantly the seed-associated epiphytes. While the epiphytic bacterial load we observed is within the range of what is observed by total aerobic plate counts on other crops such as bean and pea sprouts (Deb & Joshi, 2007), there is no published baseline data on total epiphytic microbial load of *Triticum* and *Brassica* crop seeds. We have observed mean aerobic plate counts of *c*. > 10^6^ CFU g^−1^ for *Triticum* and 10^5^–10^6^ for *Brassica* (R. Clear & T. Demeke, unpublished), a trend that is in line with our observations reported herein. An endophytic bacterial load in this same range has been reported for *B. napus* seeds (Granér *et al*., 2003). For some related crops, such as buckwheat, customers may set limits on total aerobic plate counts that are considerably lower (e.g. 5.5 log_10_ CFU g^−1^ seeds) than we observed for *Triticum* and *Brassica* (Dhillon *et al*., 2012), and lower total microbial loads are generally seen as desirable (Olaimat & Holley, 2012). The molecular methods used to estimate bacterial genomes g^−1^ seeds are unable to distinguish between viable and nonviable microbes, so estimates of total bacterial load by aerobic plate counts may be considerably lower than is determined using molecular methods. Nevertheless, our results establish a baseline epiphytic microbial load for food-grade seeds of *Triticum* and *Brassica*.

An overall total of 5477 OTU was associated with all *Brassica* and *Triticum* samples. We acknowledge that we may not have sampled sufficient numbers of representatives of each of these two genera to make definitive genus-based conclusions, and other experimental designs, such as the inclusion of more species of each genus and the inclusion of other genera may be required to fully elucidate the compositions of the *Brassica* and *Triticum* seed-associated microbiota. Nevertheless, our results establish a baseline for these crop plants and identify a core microbiota for all *Brassica* samples (215 OTU) as well as all *Triticum* samples (262 OTU). We also identified a shared microbiome among these seeds from distinct host plant genera harvested from a range of geographic locales, separated by thousands of kilometers. The existence of a shared microbiome conserved across plant genera suggests that the seed-associated microbiome is not a casually associated surface contamination but rather a selected, host-specific community, intimately associated with the host, and with potentially profound effects on seed health. These observations are consistent with previous studies of the seed-associated endophytic bacteria within *Zea* spp. (corn), wherein a microbiota was identified that is conserved in various teosinte progenitor species grown in an array of geographical locations (Johnston-Monje & Raizada, 2011). Despite these commonalities, the *Triticum* and *Brassica* seed microbiota could be distinguished based on the relative abundances of shared OTU (Fig.2).

Studies of *Zea* seed endophytes revealed a preponderance of Gammaproteobacteria including *Enterobacter*, *Pantoea* and *Pseudomonas* spp. (Johnston-Monje & Raizada, 2011). Similarly, Weiss *et al*. examined the microbiota associated with alfalfa, radish and bean sprouts and found the same genera represented, along with *Lactobacillus* (Weiss *et al*., 2007). The majority of bacterial taxa that we observed in the *Brassica–Triticum* shared microbiome included OTU that were closely related to these genera (Table S1). Many of the microorganisms we identified on the seed surface are also found in soil, suggesting a possible relationship between soil microbiota and seed-borne microorganisms. This is consistent with the fact that *Triticum* and *Brassica* seeds are sown into soils, commonly in rotation with one another. Airborne microbes are another possible source of seed-associated epiphytes. The seed microbiome included a relatively large proportion of OTU that were closely related (95–99% sequence identity) to *Pantoea agglomerans*, including 78 that were significantly differentially abundant on *Triticum* compared with *Brassica* seeds. Among the fungal OTU were several with similarity to yeasts and Ascomycetes, including *Fusarium*. While certain species of *Fusarium* are wheat pathogens, no sequences identical to known pathogens were detected on these seeds; however, given the ability for the seeds to be associated with microbes closely related to pathogens there is a clear need to monitor seed health. In addition, 18 OTU were identified that clustered with microorganisms such as *Pyrenophora*, *Alternaria* and *Leptosphaeria,* of which there are related pathogenic species that can cause grain spoilage. While none of the seeds in this study displayed any visual indication of microbial contamination/colonization, these findings demonstrate that the seed microbiome is crucial as it may harbor both beneficial and potentially pathogenic organisms.

Our data also indicate that observations of OTU abundance patterns can lead to the recognition of interactions between microbes with significant implications for the host. Relatively high levels of *Pantoea-*like OTU and significantly lower levels of *Alternaria*-like OTU were detected on *Triticum* seeds, while this relationship was reversed on *Brassica* seeds (Figs3). The reciprocal abundances of *P. agglomerans* and *Alternaria* sequences on *Triticum* and *Brassica* seeds, validated by quantitative PCR, suggested a potential antagonistic relationship between these microbes. It is well known that *P. agglomerans* can be antagonistic to *L. maculans* and other pathogens (Kempf & Wolf, 1989; Chakraborty *et al*., 1994; Kearns & Hale, 1996; Bryk *et al*., 1998; Braun-Kiewnick *et al*., 2000), but inhibition of the growth of *Alternaria* spp. by *P. agglomerans* has not been described. The fact that we identified this organism within the epiphytic microbiota of *Triticum* and *Brassica* seeds suggests that organisms with pathogen-protective effects naturally associate with seeds. By contrast, *Alternaria* spp., distinct from those detected on the seeds within this study, can cause grain safety concerns in storage due to the production of mycotoxins by specific species (Greco *et al*., 2012). These observations suggest that the *P. agglomerans* strain we identified in this study has potential as a biocontrol agent, and if applied to seeds may act to protect them from storage-associated spoilage or colonization with pathogenic microorganisms.

We have identified a remarkably conserved epiphytic microbiome on the seeds of geographically and ecologically diverse samples of two important crops. Reproducible differences in the abundances of constituents of this core microbiota were used to identify patterns that were associated with each crop type. Furthermore, this work has shown that differences in OTU abundance within and between microbiomes can be valuable clues and indicators of biological interactions among microorganisms. Finally, we demonstrated a method for simultaneous profiling of the bacteria and fungi within the epiphytic microbiota of crop seeds. These results provide a system for understanding the microorganisms that are associated with seeds, and highlight the need for a thorough understanding of these microbial communities and their importance to production and storage of healthy, high quality seeds.

## Supporting Information

**Fig. S1** Family-level taxonomic profile of *Triticum* and *Brassica* samples. **Fig. S2** Community statistics for *Triticum* and *Brassica* seed samples. **Fig. S3** Determination of the *Triticum*/*Brassica* seed-associated shared OTU with increasing paired sample size. **Fig. S4** Phylogenetic analysis of ITS sequences of fungal isolates compared with reference sequences. **Fig. S5** Interactions of selected bacterial isolates with the fungal pathogen *Leptosphaeria maculans*.

**Notes S1** Rarefaction plots.

**Table S1** List of OTU shared between *Triticum* and *Brassica* seed washes
