# Supplementary material for: Simultaneous profiling of seed-associated bacteria and fungi reveals antagonistic interactions between microorganisms within a shared epiphytic microbiome on Triticum and Brassica seeds
Source: New Phytol. 2014 Jan 21;202(2):542–53. doi: 10.1111/nph.12693 (PMC4235306; doi:10.1111/nph.12693)
Supplement: Supplementary file 1 — Fig. S1 Family-level taxonomic profile of Triticum and Brassica samples. Fig. S2 Community statistics for Triticum and Brassica seed samples. Fig. S3 Determination of the Triticum/Brassica seed-associated shared OTU with increasing paired sample size. Fig. S4 Phylogenetic analysis of ITS sequences of fungal isolates compared with reference sequences. Fig. S5 Interactions of selected bacterial isolates with the fungal pathogen Leptosphaeria maculans. [file nph0202-0542-SD1.docx]

**Supporting Information Figs S1–S5**

Simultaneous profiling of seed-associated bacteria and fungi reveals antagonistic interactions between microorganisms within a shared epiphytic microbiome on *Triticum* and *Brassica* seeds

Matthew G. Links^1,2^, Tigst Demeke^3^, Tom Gräfenhan^3^, Janet E. Hill^2^, Sean M. Hemmingsen^4^ and Tim J. Dumonceaux^1,2^

^1^Agriculture and Agri-Food Canada Saskatoon Research Centre, Saskatoon, SK, Canada; ^2^Department of Veterinary Microbiology, University of Saskatchewan, Saskatoon, SK, Canada; ^3^Grain Research Laboratory, Canadian Grain Commission, Winnipeg, MB, Canada; ^4^National Research Council Canada, Saskatoon, SK, Canada

**
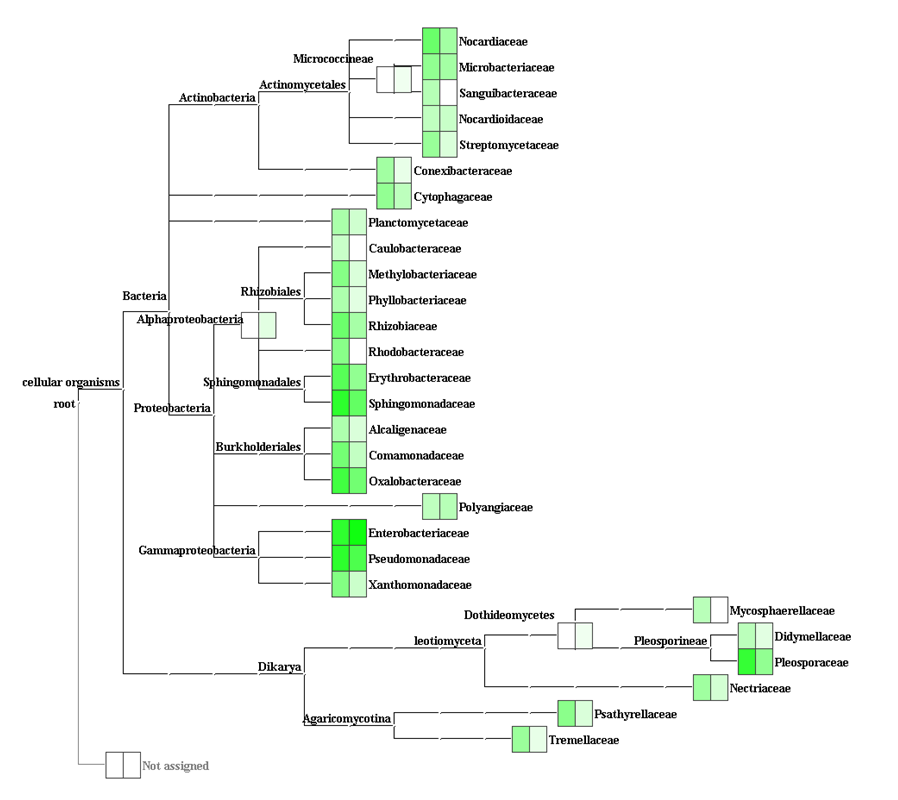
**

**Fig. S1** Family-level taxonomic assignment of reads shared among *Triticum* and *Brassica* samples. Included in this analysis were the 578 OTU that were found in at least 7/11 libraries and therefore were represented at least once in both *Triticum* and *Brassica*. MEGAN (**Mitra S, Klar B, Huson DH. 2009.** Visual and statistical comparison of metagenomes. *Bioinformatics* **25:**1849–1855) was used to analyze the nucleotide sequences of assembled OTU using BLASTn against a non-redundant version of cpnDB with the following Lowest Common Ancestor (LCA) parameters: Min support = 5; min score = 100; top percent = 20. The results are visualized as a heat map with *Brassica* read abundances on the left and *Triticum* on the right. Higher abundances are shown as darker shades of green.

**Fig. S2** Community statistics for *Triticum* and *Brassica* seed samples. (a) Simpson’s index (1/D), which measures community evenness. Error bars represent the 95% confidence intervals of the data. (b) Shannon index (H’), which is a combined measure of community richness and evenness (**Hill TCJ, Walsh KA, Harris JA, Moffett BF. 2003.** Using ecological diversity measures with bacterial communities. *FEMS Microbial Ecology* **43**: 1–11). No statistically significant differences were detected between *Brassica* and *Triticum* samples for any of these community diversity measures (Mann-Whitney rank sum test, *P* > 0.05).

a

b

**Fig. S3** Determination of the *Triticum*/*Brassica* seed-associated shared OTU with increasing paired sample size. All possible combinations of *Triticum* and *Brassica* libraries were compared at each sample size (e.g. 1 *Triticum*-1 *Brassica*; 2 *Triticum*-2 *Brassica*, *etc*.) and the number of OTU that were observed in all samples of both *Triticum* and *Brassica* was determined. The median value for each data point is shown by a horizontal line, and the outer edges represent the 10^th^ and 90^th^ percentiles of the data.

**Fig. S4** Phylogenetic analysis of ITS sequences of fungal isolates compared to reference sequences. Sequences were trimmed to the same length (570-600 bp) prior to alignment. The tree was constructed as described in the Materials and Methods section, with the percentage of 500 replicates showing identical branching/clustering patterns shown next to the nodes. The scale bar represents units of base substitutions per site. Reference sequences were obtained from GenBank, with the accession numbers indicated.


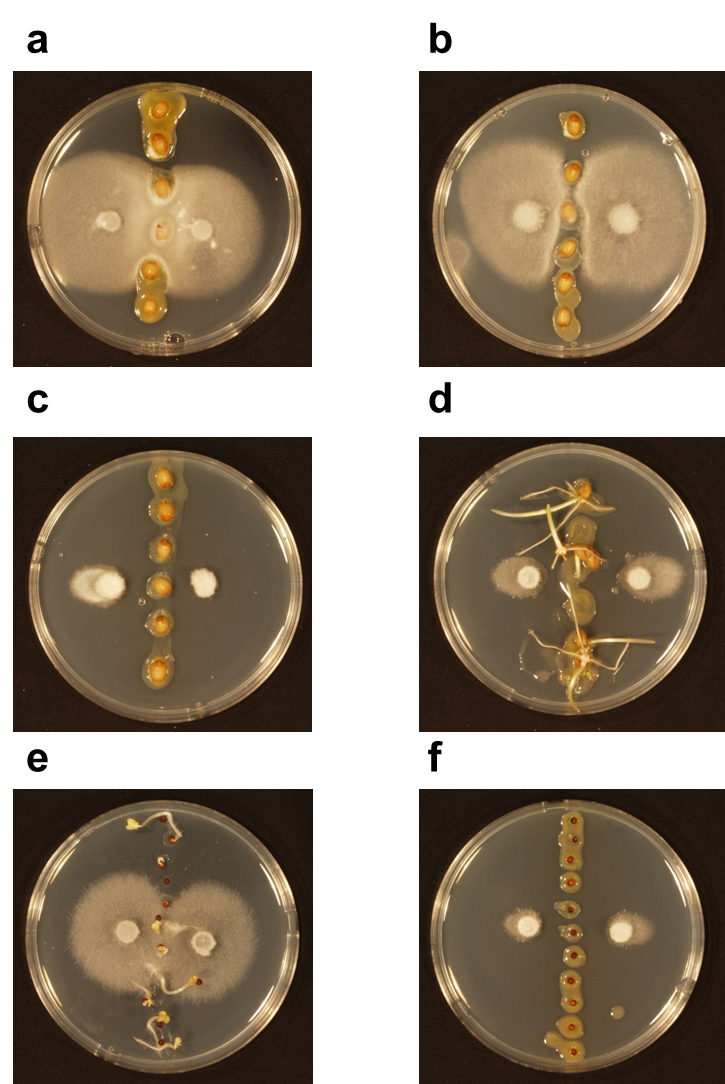
**Fig. S5** Interactions of selected bacterial isolates with the fungal pathogen *Leptosphaeria maculans*. (a) Sterilized wheat seeds colonized with bacterial isolate #8 (homogenous). (b) Same as (a), but with bacterial isolate #9 (growth cessation at point of contact). (c) Same as (a), but with bacterial isolate #4 (aversion). (d) Nonsterilized wheat seeds colonized with bacterial isolate #4. (e) Nonsterilized canola seeds. (f) Nonsterilized canola seeds colonized with bacterial isolate #4.
